# Determinants of inappropriate admissions of children to county hospitals: a cross-sectional study from rural China

**DOI:** 10.1186/s12913-019-3944-1

**Published:** 2019-02-18

**Authors:** Shi-han Lei, Yan Zhang, Hao-miao Li, Dai Su, Jing-jing Chang, Xiao-mei Hu, Qing Ye, Di Jiang, Ying-chun Chen

**Affiliations:** 1 School of Medicine and Health Management, Tongji Medical College, Huazhong University of Science and Technology, Wuhan, 430030 Hubei China; 2 Research center for Rural Health Services, Hubei Province Key Research Institute of Humanities and Social Sciences, Wuhan, 430030 Hubei China

**Keywords:** Inappropriate admission, Children, County hospital, Rural China

## Abstract

**Background:**

The incidence of inappropriate admissions in China has become the shackle of its’ service supply system. This research aims to assess the level of children’s inappropriate admissions to county hospitals in rural China and identify the characteristics and determinants of children’s inappropriate admissions.

**Methods:**

A retrospective review was conducted on data of children aged 0–14 years. A total of 771 children medical records in four county hospitals was collected by stratified random sampling in Midwestern China and was evaluated through the Rural Appropriateness Evaluation Protocol. A questionnaire survey was conducted among doctors whose names were shown in medical records. Chi-square test was used to analyse the characteristics of inappropriate admissions, and a binary logistic regression model was used to examine the determinants of inappropriate admissions.

**Results:**

Inappropriate admissions indicate that patients who could have been treated as outpatients received services as inpatients. The average rate for inappropriate admissions of children in county hospitals was 61.35%. The highest rate of inappropriate admissions was found among children aged 1–5 years (68.42%). Inappropriate admissions mostly occurred in children with respiratory diseases (72.45%), circulatory diseases (72.22%) and certain infectious diseases and parasitic diseases (70.37%). Binary logistic regression analysis showed that county, normal health status, treating department, disease, the length of hospital stay and the doctor’s self-evaluation on the understanding about the degree of the patient’s feelings were determinants for children’s inappropriate admissions.

**Conclusions:**

County hospitals have a high rate of inappropriate admissions of children. The relationship of children’s inappropriate admissions to age distribution and the insurance compensation is affected by disease and hospitalisation expenses, respectively. The determinants of children’s inappropriate admissions are directly related to the weak level of primary care services in the health service system, the initial requirements requested by children’s admission decision makers and the interests among medical institutions and doctors.

**Electronic supplementary material:**

The online version of this article (10.1186/s12913-019-3944-1) contains supplementary material, which is available to authorized users.

## Background

Inappropriate admissions indicate that patients who could have been treated as outpatients received services as inpatients [1, 2]. This phenomenon increases the average medical expenditure and waste in health resources [3]. Some studies have reported that inappropriate admissions have substantially contributed to high hospitalisation rate [4]. From 2003 to 2013, the hospitalisation rate for children aged 0–14 years in rural China increased by 2.86 times from 1.73 to 4.95% [5]. The increasing hospitalisation rate for children expanded faster than the forecast, suggesting that inappropriate admissions might occur. Several studies have indicated that children are the high-risk group for inappropriate admissions [6, 7]. In 2013, the rate of inappropriate admissions for children (aged 0–14 years) in county hospitals was 21.8%, which was higher than that for people aged 15–64 years (5.8%) and aged 65 years and older (5.6%) [7]. The number of children in China has increased with the universal two-child policy. Inappropriate admissions can cause cross-infection and other threats to children’s health [8]. Therefore, controlling the inappropriate admissions of children is important.

As a special group in society, children lack the ability to decide whether or not they should be hospitalised. Their admission often stems from their guardians or doctors [8]. National conditions, including China’s long-term one-child policy, ensure that the inappropriate admissions of children in China are different from those in other countries in some way. On the one hand, urban areas include municipalities and prefecture-level cities; rural areas include counties or county-level cities and their subordinate towns and villages. Therefore, the establishment of medical institutions in China covers municipal hospitals, county hospitals, township hospitals and village clinics. The latter three of the above-mentioned medical institutions are rural medical institutions [9]. Among them, county hospitals have the strongest capacity in providing medical services, so they play a leading role within counties. In terms of hospitalisation services, county hospitals treat more kinds of diseases than township hospitals. Meanwhile, village clinics generally do not provide inpatient services. Taking a prefecture-level city in China as an example, data show that among the pediatric inpatients classified by municipal hospitals, county hospitals, township hospitals and private hospitals, the number of hospitalised children in county hospitals accounts for 50.2% of the total number of hospitalised patients; children hospitalized in municipal hospitals and township hospitals was 39.4 and 7.3% of the total number of patients, respectively [10]. The tendency of children patients to hospitalise in county hospitals affects the rate of inappropriate admissions of children in county hospitals. On the other hand, from the perspective of service providers, doctors tend to provide extended services to be on the safe side because children cannot truly describe their feelings. This phenomenon could lead defensive medicine which influences the occurrence of inappropriate admissions [11, 12].

In recent years, the Chinese government has issued a series of policies, such as the payment reform of new rural cooperative medical system (NRCMS) and the family doctor contracting services, to guide patients to seek appropriate medical advices and prevent improper hospitalisation behaviours. However, the unreasonable demands and utilisations of inpatient services remain unresolved. Causes of this problem are not yet clear. It is necessary to identify the determinants of children’s inappropriate admissions, so as to improve the efficiency of the utilisation of hospital services, avoid unnecessary waste of medical resources, and ensure the quality and safety of children’s medical services.

Most studies on inappropriate admissions of children were based on their medical records provided by hospitals [6, 13–23]. They mainly focused on evaluating the rate of inappropriate admissions, identifying the basic characteristics and influencing factors and further analysing health policies, hospital management system and health service behaviors behind them. In terms of characteristics, studies focused on the severity of illness and diseases in hospitalised children. For instance, De Marco et al. suggested that children with influenza-like illness are likely to have inappropriate admissions [6]. On the analysis of influencing factors, Aida et al. found that the ratio of inappropriate admissions could be reduced by strengthening the relationship between hospitals and community service agencies [15]. Magdy et al. believed that the rate of inappropriate admissions of children in Kuwait was up to 40%, which can be explained by its’ free health care services and easy access to hospital services [13]. In China, a few studies were conducted about inappropriate admissions on the whole population including children were conducted. Meanwhile, the existing studies did not focus on the children in county hospitals. Given differences in the determinants of inappropriate admissions of children and other groups, factors influencing inappropriate admissions of children at the county hospitals are worthy of further investigation. Therefore, this study investigated the determinants of children’s inappropriate admissions. The research hypothesis of this study is that the appropriateness of children hospitalisation to county hospitals in rural China is related to the health policies and environments factors regarding children.

This study aims to investigate the level and determinants of the inappropriate admissions of children to provide comprehensive and targeted strategies for the managers and decision-makers in controlling this phenomenon.

## Methods

### Sample selection

We conducted this research in 2017, and the surveyed population was inpatient aged 0–14 years. In the eastern part of China, many rural areas rapidly became cities due to social development. Hence, county property attributes decreased. Therefore, this study did not include the eastern part of China in the research scope. We applied stratified random sampling and the principle of convenience in selected counties and county-level cities in China’s central (e.g. Dingyuan in Anhui Province) and western (e.g. Weiyuan and Huining in Gansu Province; Yilong in Sichuan Province) regions, which were designated as sample areas. The biggest county hospital in each county was used as the sample hospital. Table 1 shows the basic information and the service provision of the sample county hospitals in the year before this study was carried out.

**Table 1:** The fundamental state of the sample county hospitals

Index	County hospital in Dingyuan	County hospital in Weiyuan	County hospital in Huining	County hospital in Yilong
Designated bed capacity	845	350	400	750
Number of actually available bed	880	450	652	780
Number of on-post staff	1233	328	951	851
Among them:				
Doctors	319	81	157	198
Registered nurses	499	49	483	376
Frequency of outpatient and emergency visits	531.5 thousand person	101.4 thousand person	228.4 thousand person	278.3 thousand person
Number of discharged patients	62.1 thousand person	14.2 thousand person	28 thousand person	29.4 thousand person

We believed that the NRCMS in different regions affected the inappropriate admissions for residents. Thus we also collected the compensation policies for NRCMS in the selected regions. Table 2 shows how the patients from the four counties benefited from the reimbursement policies of NRCMS. These counties shared the same financing policies in 2017. Reimbursement took two forms, namely, outpatient and inpatient reimbursements. The outpatient service in county hospitals was not covered. The reimbursement of inpatients ranged from 75 to 85% of the total reimbursements, and the deductible line was between 400 and 650 RMB. Overall, the NRCMS hospitalisation benefits were high in all regions.

**Table 2:** NRCMS reimbursement polices in the survey areas in 2017

County		Dingyuan	Weiyuan	Huining	Yilong
Payment		Global budget	Payment for single disease	Payment for single disease	Global budget
Reimbursement of Outpatient	Reimbursement Ratio	45% for county hospitals	60% for county hospitals	60% for county hospitals	60% for county hospitals
		50% for village clinic	60% for village clinic	60% for village clinic	60% for village clinic
	Annual Payment Limitation	150 RMB	150 RMB	300RMB	120RMB
Reimbursement of Inpatient	The Low Limit of Pay Line	620 RMB for county hospitals	500 RMB for county hospitals	500 RMB for county hospitals	400 RMB for county hospitals
		150 RMB for township hospitals	150 RMB for township hospitals	150 RMB for township hospitals	100 RMB for township hospitals
	Reimbursement Ratio	85% for county hospitals	70% for county hospitals	80% for county hospitals	75% for county hospitals
		90% for township hospitals	80% for township hospitals	90% for township hospitals	90% for township hospitals
	Single payment limitation	**-**	15,000 RMB for county hospitals	20,000 RMB for county hospitals	-
			3,000 RMB for township hospitals	3,000 RMB for township hospitals	
	Annual Payment Limitation	200,000 RMB	80,000RMB	80,000RMB	180,000RMB

### Sample size estimation

According to previous studies, the estimated inappropriate admission rate *P* is 40%, the expected error δ is 0.035 and the confidence level α is 0.05 in the sampling calculation:

$$ \mathrm{N}={\mathrm{Z}}_{\alpha /2}^2\times P\left(1-P\right)/{\updelta}^2={1.96}^2\times 0.40\times \left(1-0.40\right)/{0.035}^2=753 $$

Based on the population in the surveyed areas and the scale of operation of the surveyed hospitals, 220 hospitalised children medical records were selected in Dingyuan county of Anhui Province and Yilong of Sichuan Province and 175 hospitalised children medical records were selected in Weiyuan and Huining County of Gansu Province. Children were admitted in departments such as paediatrics, surgery, orthopaedics and internal medicine. Our study selected number of medical records in each department relative to the proportion of children in the department. After the non-evaluable medical records were removed, 771 medical records of inpatients in four counties were finally obtained.

### Records appropriateness evaluation

Considering the actual situation of China, 771 admission records were collected at the beginning and identified through the improving rural appropriateness evaluation protocol (RAEP) which was culturally suitable and widely used in this country (Additional file 1). There were three specially trained experts evaluated the medical records collected, all of whom were Ph.D. and involved in health policy research more than five years. Experts who owned the rich of research experience and deep understanding of existing services in rural China enabled to make the fair judgment on records with strict accordance with standards. Furthermore, each record was examined by two judges to ensure the quality of the medical records. If two judges had different opinions, the medical record would be sent to a third party. We obtained the results after a comprehensive evaluation. The RAEP standard was based on the value of the medical records. Therefore, all RAEP related indexes were extracted firstly. The actual values were then compared with the standard values. If an index value corresponded to the standard value, the admission record was appropriate. If all the relevant values of the medical record did not conform to the evaluation table criteria, the record was an inappropriate admission.

### Research content

First, information, including personal and hospitalisation information of patients, was collected from the inpatient medical records. The patient’s personal information included the patient number, gender, age, normal health status and whether to get the insurance compensation. Other inpatient information included the admission department, name of the doctor in charge, day of the week of admission, emergency admission, admission and discharge diagnosis, test results, operation conditions and the length of hospital stay (LOS). After obtaining the hospitals’ consent, we conducted a questionnaire survey of the doctors based on the doctor’s name displayed in the inpatient medical records. The doctors’ information involved age, educational background, their sense of the degree to which doctors believe they have autonomy in clinical decision-making, the degree to which doctors believe that they can understand the patient’s feelings, the degree to which they think the tension in doctor-patient relationship (Additional file 2).

### Statistical analysis

Medical record information of inpatients was recorded through Epidata 3.2. Diseases were classified using the international classification of diseases 10th Edition (ICD-10).

The characteristics of patients inappropriately admitted to county hospitals were analysed using Pearson’s chi-square test in IBM SPSS Statistics 22.0. Given that Weiyuan County and Huining County were affiliated to the same province and considering the possible clustering between the regions, this study used the generalised estimating equation to build the model and set the job-related matrix type to unstructured firstly. However, in the correlation matrix, the main diagonal element was close to 1, and the remaining elements were near 0. Hence, no clustering occurred between the sampling regions. A binary logistic regression model was used to analyse the determinants of inappropriate admissions of children. The patient’s admission appropriateness (appropriate =0, inappropriate =1) was used as the dependent variable, whereas county, sex, age, compensation for medical insurance, normal health status, admission department, day of the week of admission, disease, the admission route (admitted emergency), LOS and information of doctors mentioned in the research content were the independent variables. Backward stepwise was used to establish the regression model as follows:

$$ Logit(P)=\mathit{\ln}\left(P/1-P\right)={\beta}_0+{\beta}_1 County+{\beta}_2 Normal\ health\ status+{\beta}_3 Department+{\beta}_4 Disease+{\beta}_5 LOS+{\beta}_6{Doctor}^{\prime }s\ \mathrm{understanding}\ {\mathrm{of}\ \mathrm{patient}}^{\prime}\mathrm{s}\ \mathrm{feelings}+\varepsilon . $$

*P* represents the probability of inappropriate admission for children, *β*_0_ is the constant term for the model and *ε* represents an uncontrollable random error term.

## Results

### Basic composition of Children’s inpatients and their doctors

The first column in Table 3 shows the children’s admission data: 61.09% were male, the average age was 4.25 years old (age range: 0–14 years old), 91.83% were patients who had gotten compensation after purchasing the NRCMS, and 89.11% were in good normal health. Most of the admissions were non-emergency, and the children with respiratory diseases accounted for 54.6% of the total number of children. The range of LOS was 1–33 days with average and median of 4.97 and 4.45 days, respectively.

**Table 3:** The Appropriation Distribution Characteristics about Children Inpatients in County Hospitals According to the Medical Records

Variable	N (column%)	Appropriate	Inappropriate	χ2	*P*
		N (column%)	N (column%)		
All	771(100.00)	298(38.65)	473(61.35)		
County					
Dingyuan	219(28.40)	63(28.77)	156(71.23)	68.083	< 0.001
Weiyuan	166(21.53)	90(54.22)	76(45.78)		
Huining	171(22.18)	95(55.56)	76(44.44)		
Yilong	215(27.89)	50(23.26)	165(76.74)		
Gender of patients					
Male	471(61.09)	181(38.43)	290(61.57)	0.025	0.874
Female	300(38.91)	117(39.00)	183(61.00)		
Age of patients, (years)					
Less than 1	93(12.06)	52(55.91)	41(44.09)	34.947	< 0.001
1–5	437(56.68)	138(31.58)	299(68.42)		
6–9	151(19.58)	56(37.09)	95(62.91)		
10–14	90(11.67)	52(57.78)	38(42.22)		
Mean (SD)	4.25(3.70)				
Whether patients get medical insurance compensation					
Yes	708(91.83)	276(38.98)	432(61.02)	0.403	0.526
No	63(8.17)	22(34.92)	41(65.08)		
Normal heath of patients					
Good	687(89.11)	242(35.23)	445(64.77)	31.204	< 0.001
General	84(10.89)	56(66.67)	28(33.33)		
Department					
Paediatrics	654(84.82)	212(32.42)	442(67.58)	70.661	< 0.001
Others	117(15.18)	86(73.50)	31(26.50)		
Day of the week of admission					
Saturday- Sunday	196(25.42)	73(37.24)	123(62.76)	0.219	0.64
Monday-Friday	575(74.58)	225(39.13)	350(60.87)		
Disease category					
Respiratory disease	421(54.6)	116(27.55)	305(72.45)	95.079	< 0.001
Digestive disease	94(12.19)	37(39.36)	57(60.64)		
Injury and poisoning	42(5.45)	30(71.43)	12(28.57)		
Conditions originating	56(7.26)	43(76.79)	13(23.21)		
in perinatal period					
Infectious Diseases	54(7.00)	16(29.63)	38(70.37)		
Symptoms and signs	21(2.72)	10(47.62)	11(52.38)		
Circulatory disease	18(2.33)	5(27.78)	13(72.22)		
Others	65(8.43)	41(63.08)	24(36.92)		
Admitted emergency					
Yes	611(79.25)	255(41.73)	356(58.27)	11.808	0.001
No	160(20.75)	43(26.88)	117(73.13)		
LOS, (days)					
Less than 5	400(51.88)	119(29.75)	281(70.25)	40.007	< 0.001
5–8	293(38.00)	128(43.69)	165(56.31)		
More than 9	78(10.12)	51(65.38)	27(34.62)		
Mean (SD)	4.97(3.43)				
Median	4.45				

The first column in Table 4 shows the doctor status corresponding to each medical record: 42.41 and 79.12% of records indicate that the pediatricians were greater than or equal to 46 years old and they had bachelor’s degree or above. In most cases, doctors believed that they could sense patients’ feelings and that they had a moderate degree of autonomy in clinical decision-making. 47.21% of the records show that doctors thought the existing doctor-patient relationship was moderate.

**Table 4:** The Appropriation Distribution Characteristics about Children Inpatients in County Hospitals under the Perception of Doctors

Variable	N (column%)	Appropriate	Inappropriate	χ2	*P*
		N (column%)	N (column%)		
Age of doctors, (years)					
Less than 36	207(26.85)	92(44.44)	115(55.56)	6.190	0.045
36–45	237(30.74)	95(40.08)	142(59.92)		
46 years old and above	327(42.41)	111(33.94)	216(66.06)		
Educational background of doctors					
Junior college and below	161(20.88)	60(37.27)	101(62.73)	0.164	0.685
Bachelor degree or above	610(79.12)	238(39.02)	372(60.98)		
The degree to which doctors believe they have autonomy in clinical decision-making					
Low	115(14.92)	48(41.74)	67(58.26)	1.118	0.572
Moderate	355(46.04)	140(39.44)	215(60.56)		
High	301(39.04)	110(36.54)	191(63.46)		
The degree to which doctors believe they can understand the patient’s feelings					
Low	159(20.62)	56(35.22)	103(64.78)	1.085	0.581
Moderate	577(72.24)	229(39.69)	348(60.31)		
High	35(4.54)	13(37.14)	22(62.86)		
The degree to which doctors believe tensions between doctors and patients					
Low	184(23.87)	91(49.46)	93(50.54)	12.777	0.002
Moderate	364(47.21)	123(33.79)	241(66.21)		
High	223(28.92)	84(37.67)	139(62.33)		

### Characteristics of children inappropriately admitted to county hospitals

Table 3 also shows the characteristics of the patients. A total of 61.35% of the records from the four sample areas indicated inappropriate admissions. The incidence rate varied in different regions (*P* < 0.001) and ranged from 40 to 80%. The Yilong County of Sichuan province had the highest rate of inappropriate admissions.

In the distribution of individual characteristics, 1- to 5-year-old preschool children and 6- to 9-year-old primary school children were likely to be inappropriately admitted (*P* < 0.001). The children with good health and in normal status were likely to have inappropriate admissions (*P* < 0.001). No statistical difference was found in gender and inappropriate admissions, medical insurance compensation and inappropriate admissions. In terms of the distribution of inpatient characteristics, the children hospitalised in paediatrics were more likely to experience inappropriate admission than those hospitalised in other departments (*P* < 0.001). Inappropriate admission was concentrated in the children with respiratory, circulatory, infectious and digestive diseases (*P* < 0.001). The children admitted in emergency (*P* = 0.001) and those with short LOS (*P* < 0.001) were likely to have inappropriate admission. No statistical difference in the day of the week of admission was found between the two groups.

Table 4 shows that patients treated by doctors over 46-year-old were more likely to be hospitalised inappropriately than those treated by doctors under 45-year-old (*P* = 0.045). Patients treated by doctors who thought the doctor-patient relationship moderate were more likely to be inappropriately admitted (*P* = 0.002). There is no significant difference among groups on the degree of doctor’s educational backgrounds, the doctors’ self-determination in diagnosis and their understanding of patients’ feelings to children’s inappropriate admissions.

### Probabilistic model of patients in children inappropriately admitted to county hospitals

The binary logistic regression model was used to analyse the determinants of inappropriate admissions of children in county hospitals. With the minimum value as the reference level, the first and ninth variable selection results are shown in Table 5.

**Table 5:** Logistic regression analysis of inappropriate admission of children inpatients (*n* = 771)

Variable	AOR	95% CI
Step-1		
County (Dingyuan)		
Weiyuan	0.685	0.355–1.321
Huining	0.506	0.275–0.929
Yilong	2.116	1.056–4.239
Gender of patients (Male)	0.976	0.686–1.389
Age of patients, (years) (Less than 1)		
1–5	1.688	0.920–3.098
6–9	1.472	0.724–2.995
10–14	1.204	0.539–2.690
Whether patients get insurance compensation (Yes)	1.275	0.663–2.452
Normal heath of patients (Good)	0.562	0.309–1.024
Department (Paediatrics)	0.185	0.094–0.363
Day of the week of admission (Saturday- Sunday)	0.685	0.459–1.023
Disease category (Respiratory disease)		
Digestive disease	1.248	0.686–2.272
Injury and poisoning	0.346	0.142–0.844
Conditions originating in perinatal period	0.245	0.115–0.523
Infectious Diseases	1.053	0.533–2.082
Symptoms and signs	0.458	0.172–1.218
Circulatory disease	1.304	0.341–4.979
Others	0.556	0.276–1.120
Admitted Emergency (Yes)	1.095	0.654–1.834
LOS, (days) (Less than 5)		
5–8	0.634	0.432–0.932
More than 9	0.505	0.266–0.958
Age of doctors, (years) (Less than 36)		
36–45	1.646	0.929–2.915
More than 46	1.264	0.756–2.113
Educational background of doctors (Junior college and below)	0.994	0.580–1.703
The degree to which doctors believe they have autonomy in clinical decision-making (Low)		
Moderate	0.632	0.307–1.301
High	0.904	0.492–1.662
The degree to which doctors believe they can understand the patient’s feelings (Low)		
Moderate	0.555	0.317–0.971
High	2.156	0.789–5.889
The degree to which doctors think tensions between doctors and patients (Low)		
Moderate	0.823	0.433–1.567
High	0.654	0.297–1.440
Constant	5.930	
Step-9		
County (Dingyuan)		
Weiyuan	0.597	0.346–1.031
Huining	0.468	0.281–0.777
Yilong	1.752	1.034–2.969
Normal heath (Good)	0.545	0.305–0.974
Department (Paediatrics)	0.173	0.092–0.324
Day of the week of admission (Saturday- Sunday)	0.690	0.465–1.024
Disease category (Respiratory disease)		
Digestive disease	1.305	0.728–2.339
Injury and poisoning	0.401	0.165–0.970
Conditions originating in perinatal period	0.215	0.104–0.446
Infectious Diseases	1.058	0.539–2.077
Symptoms and signs	0.536	0.206–1.396
Circulatory disease	1.219	0.335–4.438
Others	0.553	0.279–1.098
LOS, (days) (Less than 5)		
5–8	0.642	0.440–0.936
More than 9 days	0.501	0.266–0.945
The degree to which doctors believe they can understand the patient’s feelings (Low)		
Moderate	0.697	0.446–1.091
High	2.302	0.862–6.149
Constant	6.694	

Based on the logistic regression analysis, the major determinants of inappropriate admissions of children to county hospitals included county, normal health status, treating department, disease LOS and the doctor’s self-evaluation on the understanding about the degree of the patient’s feelings. With Dingyuan County as the control group, the possibility of inappropriate admission in Weiyuan (OR = 0.597) and Huining (OR = 0.468) was significantly low. The children with poorer normal health were more likely to have appropriate admission (OR = 0.545) than the children with good health. The children hospitalised in other departments had lower inappropriate hospitalisation rate than those in paediatric department (OR = 0.173). Compared with the children with respiratory diseases, children with certain conditions originating from perinatal period had a lower likelihood of inappropriate admissions (OR = 0.215). Moreover, the children with LOS of 5–8 days (OR = 0.642) and 9 days or more (OR = 0.501) or whose doctor believed that he/she could understand the patient’s feelings moderately (OR = 0.697) had the lower risk of inappropriate admissions than those others.

## Discussion

### Inappropriate admissions of children in county hospitals in rural China

In 2017, the rate of inappropriate admissions of children aged 0–14 years in Chinese county hospitals was 61.35%, which was higher than the rate in other countries such as 40.7% in Kuwait (2012), 29% in Canada (1993) and 23% in Sweden (2004) [13, 20, 21]. Based on the previous statistics, the differences in inappropriate admission rates were due to the differences in the specific age groups of children in each study and the differences in health service systems among these countries. Firstly, China has not yet adopted a mandatory system of primary care, so patients have high autonomy in the choice of institutions for medical treatment [23]. Secondly, China is implementing family doctor contracting services, which usually means that a service team composed of township hospitals and village clinic doctors should provide basic public health services and medical guidance to rural residents to improve their health behaviors and to clarify medical care [24]. However, most of the primary medical institutions in China focus on the provision of basic public health services projects rather than guiding patients to seek medical service reasonably. This easily leads patients to select a relatively high-quality county hospital for treatment, rather than choosing the primary medical institution that also be capable to diagnose and treat the disease.

The 61.35% rate of children’s inappropriate admission in this study is higher than that in previous (2013). This change is due to the difference between the policy environments of each study time and the children’s characteristics. For one thing, changes in policy environments may lead to an increase in the rate of inappropriate admissions through changes in the interest-driven mechanism of medical institutions. For another, children have vulnerabilities, and their diseases differ from those of other populations [25]. Risk aversion refers to the strategy of taking the initiative to abandon or change measures to avoid the risks when the possibility of risk loss is high. Therefore, when children’s diseases symptoms are mainly characterized by long-term cold, fever, diarrhoea and abdominal pain, the parents tend to have their children hospitalised. Moreover, because relations between doctors and patients are more strained than before. Doctors will consider that parents are more sensitive to the treatment mood of children, that is, out of concern for children, parents are more anxious when their child is sick, and that the instability of outpatient infusion treatment, i.e. the side effects of infusion treatment are great. They may admit children who do not need hospitalisation. This phenomenon also increases the possibility of inappropriate admissions.

### Characteristics of children who were hospitalised inappropriately

The characteristics including gender, disease, treating department, admission in the emergency, the day of the week of admission and LOS in the hospital were consistent with those in the previous research results [13–23]. Except for gender, the day of the week of admission, educational background of doctors, doctors’ view on their decision-making power and their understanding of patients’ feelings, the other variables had remarkable differences in the appropriateness of hospitalisation of the children. In the present study, the rate of inappropriate admission of 0- to 1-year old (infant stage) is low, which occurs because infants are likely to be hospitalised for preterm birth, congenital problems and infectious diseases [16]. Zhang Yan et al. pointed out that the NRCMS reimbursement design directly determines the choice of medical service and leads to the patient’s moral hazard. In this study, however, there is no correlation between obtaining medical insurance compensation and rationality of children’s admission [22, 26]. Previous studies also showed that when inpatient and outpatient costs were similar, the patients decided to hospitalise [27]. Therefore, the low cost of hospitalisation for children, in certain degree, can explain the relationship between medical compensation and children’s inappropriate admissions.

### Determinants of Children’s patients who were hospitalised inappropriately

County, normal health status, department, disease, LOS and the degree to which doctors believe they can understand the patient’s feelings significantly affect the appropriateness of the children’s hospitalisation in county hospitals in rural China (Table 5). International experience shows that the mode of medical insurance payment determines the mode of operation of medical providers, thereby affecting the inappropriate admissions [28–30]. Therefore, the differences of NRCMS payment methods in the investigated areas led to the discrepancies among county hospitals. In this study, the payment method in Dingyuan and Yilong is the global budget, which emphasises reducing the inappropriate services through total amount control. This may cause the county hospital to inhale a large number of patients who should be hospitalised in the township hospital since the county hospital is the centre of the rural medical system. The payment method in Weiyuan and Huining is payment for the single disease, which emphasises the reduction of inappropriate inducement by controlling single diseases. However, there may be doctors who decompose hospitalised patients with comorbidities, resulting in inappropriate admissions. Therefore, the medical insurance department should increase supervision and scientifically promote the reform of medical insurance fund payment.

Because of lacking the mastery of medical expertise, children with poorer normal health status are less prone to inappropriate admissions. On the one hand, when children who rarely get sick suddenly become ill, their parents will have excessive care, which may result in excessive demand for children’s inpatient services [8]. On the other hand, some children who are not in good normal health had experienced medical treatments. Parents of these children have an increased understanding of reasonable treatment methods, thus reducing the generation of inappropriate services. As a result, we can reduce the occurrence of inappropriate admissions by strengthening the management of children’s health and guiding parents to seek correct medical service. Consistent with most studies, the treating department has an effect on inappropriate admissions [31]. Paediatric departments are likely to have inappropriate admissions due to the internal management of hospitals. The management problems are reflected in the admission criteria and the incentive mechanism. For one thing, most of the children hospitalised in other departments are surgical patients; hence, their needs for hospitalisation are easy to generate. For another, given that the assessment of doctors is linked to the number of admissions, the doctor’s personal salary is linked to the business volume, and the admission criteria are not unified. Under the background of information asymmetry between doctors and patients, doctors are prone to induce demand and thus affect hospitalisation [32–34]. Therefore, we should pay attention to the management of paediatric departments and define the admission criteria for future management. Disease to inappropriate admissions is consistent with the previous research results. The main diseases of children are common diseases, such as bronchopneumonia and upper respiratory tract infection [6]. Most of these diseases have not reached the standards of admission; hence, the rate of inappropriate admission is high. Meanwhile, the outpatient department has different treatments and control levels for different system diseases. For example, cases originated from the perinatal period and child nursing-sensitive situations are not well controlled in the outpatient department. Hospitalisation is more appropriate than outpatient care under these circumstances [35]. The inpatient’s LOS affects the occurrence of inappropriate admissions. Accordingly, the inappropriate admission rate can be reduced by controlling the first diagnosis at township hospitals or community-based medical institutions.

### Limitations

The shortcomings of this study are as follows: Firstly, this study is a cross-sectional study due to the limited investigation time. A panel data structure for the children admitted inappropriately is impossible to establish. The conclusions may not be comprehensive enough. Secondly, the international standard of Paediatrics Appropriateness Evaluation Protocol was established for the appropriateness of children’s admission, but to judge whether or not these standards are applicable to county hospitals in rural China is impossible. This study takes RAEP to evaluate the hospitalisation records of children in county hospitals in China, so there may be deviations in the criteria for different populations. Thirdly, the content of the doctor’s survey is not rich enough, and further research is needed in the future.

## Conclusions

According to RAEP, the incidence of inappropriate admission of children to county hospitals in Chinese rural areas is high. This high incidence is directly related to the weak level of primary care services in the health service system, the initiative requirements of the children’s admission decision makers, the interests of the medical institutions and doctors. This study found that the government can control inappropriate admissions of children from the following aspects: 1) Improve the rural health service system. The inappropriate admissions of children can be controlled by improving children’s disease diagnosis and treatment in township hospitals and the level of primary medical treatment and two-way referrals. 2) Strengthen the leading role of medical insurance. Firstly, we should integrate the interests of medical service providers in the reformation and promotion of the mode of payment. Secondly, the role of medical insurance in the admission supervision of medical institutions should be strengthened. The regulatory content includes a clear admission standard. 3) Reinforce the health management of children. The primary medical staff can guide the parents’ effective medical treatment by educating the children’s guardians on the prevention and treatment of children’s common diseases to reduce the inappropriate admission rate.

## Additional files

Additional file 1: AEP criteria for county hospitalisation. Shows the checklist about the adjusted AEP criteria for county hospitals which was published on International Journal of Environmental Research and Public Health, May 2018 [22]. In addition, the AEP for township hospitals was published on BMC Health Services Research in December, 2014 [26]. (DOCX 16 kb)

Additional file 2: Questionnaire for doctors in county hospitals. This is a questionnaire for doctors in county hospitals, in order to conduct research on the appropriateness of hospital admissions to children. The questionnaire is divided into three parts: (1) The basic situation of doctors; (2) The doctors’ cognition of their work and working environments; (3) The effects between doctors’ cognition and their treatment behaviours. (DOCX 22 kb)

## Abbreviations

LOS: Length of hospital stay
NRCMS: New rural cooperative medical system
RAEP: Rural appropriateness evaluation protocol
