# Supplementary material for: Determinants of inappropriate admissions of children to county hospitals: a cross-sectional study from rural China
Source: BMC Health Serv Res. 2019 Feb 18;19:126. doi: 10.1186/s12913-019-3944-1 (PMC6378739; doi:10.1186/s12913-019-3944-1)
Supplement: Supplementary file 1 — AEP criteria for county hospitalisation. Shows the checklist about the adjusted AEP criteria for county hospitals which was published on International Journal of Environmental Research and Public Health, May 2018 [22]. In addition, the AEP for township hospitals was published on BMC Health Services Research in December, 2014 [26]. (DOCX 16 kb) [file 12913_2019_3944_MOESM1_ESM.docx]

**Additional file 1: AEP criteria for county hospitalisation**

| **A.** | **Needed medical service** |
| --- | --- |
| 1. | Need surgery or follow-up treatment within 24 hours: (1) local anesthesia or general anesthesia; and/or (2) instruments or other facilities that are only available for hospitalised patients (angiography, visceral biopsy) |
| 2. | Treatment with varying dosage or drug on a regular basis under direct medical supervision |
| 3. | Calculation of intake and output volume |
| 4. | Operation to be conducted on the following day in the operating room, detailed pre-operative consultation or evaluation on the day of admission |
| 5. | Main surgical incision and drainage nursing |
| 6. | Quarantined patients |
| 7. | Bedside electrocardiogram (ECG) monitoring or testing vital signs at least every 2 hours |
| 8. | Stopping (at least once every 8 hours) or continuing oxygen inhalation |
| 9. | Referral of post-operative recovery |
| **B.** | **Severity of illness** |
| 1. | Continuous fever>38.0°C for more than 5 days |
| 2. | Acute confusion (coma or adiaphoria) |
| 3. | Severe anomaly in electrolyte or blood and vigor, showing the following situations: (1) Na<123 mEq/L or>156 mEq/L; (2) K<2.5 mEqt/L or>6.0 mEq/L; (3) HCO3 <20 mEq/L or>36 mEq/L; and (4) arterial blood pH<7.30 or>7.45 |
| 4. | Loss of sight or hearing for 48 hours |
| 5. | Loss of activity in any part of the body for 48 hours |
| 6. | Excretion disorder or absence of intestinal peristalsis in the past 24 hours |
| 7. | Active bleeding |
| 8. | Needing blood transfusion because of bleeding |
| 9. | Mental disorders caused by non-alcohol dependence |
| 10. | Viscera removal or surgical wound dehiscence |
| 11. | Pulse less than 50 times or greater than 140 times per minute |
| 12. | Abnormal blood pressure: systolic blood pressure<90 mmHg or>200 mmHg and/or diastolic blood pressure<60 mmHg or>120 mmHg |
| 13. | Ventricular fibrillation or acute myocardial ischemia shown by electrocardiogram (ECG) report or course log |
| 14. | Acute blood disorder, severe medium-sized leukopenia, thrombocytopenia, leukocytosis, erythrocytosis, thrombocytosis or hemolysis-resulted symptoms |
| 15. | Progressive acute neurological disorders |
| 16. | Soft tissue injuries affecting basic self-care |
| 17. | Acute myocardial infarction or cerebrovascular accident (stroke) |
| 18. | Spinal cord lesions |
| 19. | Lung infection above 40% or leafy lesions according to X-ray examination |
| 20. | Hyperemesis or acute pain at acute attack by chronic diseases |
| 21. | Burns occurred in specific areas |
