# Supplementary material for: Determinants of inappropriate admissions of children to county hospitals: a cross-sectional study from rural China
Source: BMC Health Serv Res. 2019 Feb 18;19:126. doi: 10.1186/s12913-019-3944-1 (PMC6378739; doi:10.1186/s12913-019-3944-1)
Supplement: Supplementary file 2 — Questionnaire for doctors in county hospitals. This is a questionnaire for doctors in county hospitals, in order to conduct research on the appropriateness of hospital admissions to children. The questionnaire is divided into three parts: (1) The basic situation of doctors; (2) The doctors’ cognition of their work and working environments; (3) The effects between doctors’ cognition and their treatment behaviours. (DOCX 22 kb) [file 12913_2019_3944_MOESM2_ESM.docx]

**Additional file 2: Questionnaire for doctors in county hospitals**

Dear doctor,

In order to conduct research on the appropriateness of hospital admissions to children, we need to collect your information. The information in this survey will be kept confidential for you, and the information collected will only be used for research purposes. We are very grateful to you for taking the time to fill out this form during your busy work, and thank you for your support and understanding of the research work.

Yours sincerely,

School of Medicine and Health Management, Tongji Medical College, Huazhong University of Science and Technology

Here are the specific questions you need to answer：

A. Basic individual information

|  | Item contents | Your answer |
| --- | --- | --- |
| A1 | Your name: |  |
| A2 | The name of the hospital where you work:  (a) County hospital in Dingyuan (b) County hospital in Weiyuan  (c) County hospital in Huining (d) County hospital in Yilong |  |
| A3 | Your Department: (a) Paediatrics (b) Others |  |
| A4 | Your gender: (a) Male (b) Female |  |
| A5 | Your age: (a) Less than 36 (b) 36-45 (c) 46 years old and above |  |
| A6 | How many years have you worked as a doctor? |  |
| A7 | Your educational background:  (a) Junior college and below (b) Bachelor degree or above |  |
| A8 | Your total income last year was RMB |  |

B. Your cognition of the work and working environments

|  | Item contents | Low | Moderate | High |
| --- | --- | --- | --- | --- |
| B1 | The degree to which you believe you have autonomy in clinical decision-making |  |  |  |
| B2 | The degree to which you believe you can understand the patient's feelings |  |  |  |
| B3 | The degree to which you believe tensions between doctors and patients |  |  |  |
| B4 | The rationality degree of the examination linking personal income to personal business volume |  |  |  |
| B5 | The degree to which you think the doctor’s occupational risk |  |  |  |

Note: Please read each statement and tick the appropriate options according to your actual situation.

C．Effects between your cognition and treatment behaviours

|  | Item contents |
| --- | --- |
| C1 | What do you think are the main factors that affect your decision on patients’ hospital admissions?  **Your answer：** |
| C2 | What do you think of your hospital's medical quality management system? Has it affected your treatment behavior? What are your suggestions for improvement?  **Your answer：** |
| C3 | What is the salary system of your institution? Has it affected your treatment behavior? What are your suggestions for improvement?  **Your answer：** |
| C4 | What do you think of the implementation of admission standards in your hospital? What is the impact on your treatment behaviors?  **Your answer：** |
| C5 | What do you think of the current doctor-patient relationship? Does this affect the admission of your patients?  **Your answer：** |
